# Population-specific material properties of the implantation site for transcatheter aortic valve replacement finite element simulations

**DOI:** 10.1016/j.jbiomech.2018.02.017

**Published:** 2018-04-11

**Authors:** Giorgia M. Bosi, Claudio Capelli, Mun Hong Cheang, Nicola Delahunty, Michael Mullen, Andrew M. Taylor, Silvia Schievano

**Affiliations:** a Centre for Cardiovascular Imaging, UCL Institute of Cardiovascular Science & Great Ormond Street Hospital for Children, London, UK; b Cardiovascular Engineering Laboratory, UCL Mechanical Engineering, London, UK; c Barts Health NHS Trust, University College London Hospital, London, UK

**Keywords:** Transcatheter aortic valve replacement, Population-specific material parameters, Finite element modelling, Implantation site mechanical response

## Abstract

Patient-specific computational models are an established tool to support device development and test under clinically relevant boundary conditions. Potentially, such models could be used to aid the clinical decision-making process for percutaneous valve selection; however, their adoption in clinical practice is still limited to individual cases. To be fully informative, they should include patient-specific data on both anatomy and mechanics of the implantation site. In this work, fourteen patient-specific computational models for transcatheter aortic valve replacement (TAVR) with balloon-expandable Sapien XT devices were retrospectively developed to tune the material parameters of the implantation site mechanical model for the average TAVR population.

Pre-procedural computed tomography (CT) images were post-processed to create the 3D patient-specific anatomy of the implantation site. Balloon valvuloplasty and device deployment were simulated with finite element (FE) analysis. Valve leaflets and aortic root were modelled as linear elastic materials, while calcification as elastoplastic. Material properties were initially selected from literature; then, a statistical analysis was designed to investigate the effect of each implantation site material parameter on the implanted stent diameter and thus identify the combination of material parameters for TAVR patients.

These numerical models were validated against clinical data. The comparison between stent diameters measured from post-procedural fluoroscopy images and final computational results showed a mean difference of 2.5 ± 3.9%. Moreover, the numerical model detected the presence of paravalvular leakage (PVL) in 79% of cases, as assessed by post-TAVR echocardiographic examination.

The final aim was to increase accuracy and reliability of such computational tools for prospective clinical applications.

## 1 Introduction

Patient-specific computational models of cardiovascular procedures allow virtual simulation of the interaction between devices and the specific individual implantation site, taking into account anatomical and physiological information from the subject (Taylor and Figueroa, 2009). These models are becoming an important tool to support cardiovascular device development, in particular for testing new designs in clinically relevant boundary conditions (Schievano et al., 2010b), but also as a clinical pre-procedural assessment methodology to prospectively aid the decision making process (Bosi et al., 2015, Cosentino et al., 2015, Schievano et al., 2010a, Wang et al., 2015). However, use of such methods in clinical practice is still limited to individual cases, mainly due to lack of large scale validation studies and the need for more accurate methodologies to capture the patient-specific mechanical response to device deployment. Indeed, whilst cardiovascular imaging enables accurate representation of the 3D anatomy, current techniques do not allow acquisition of the patient-specific *in vivo* mechanical characteristics. Response to device deployment depends not only on the material properties of the implantation site itself, but also on the presence of surrounding structures (Kim et al., 2013), thus limiting in some contexts the value of *ex-vivo* data from arterial tissue (Avril et al., 2010, Badel et al., 2011, Cabrera et al., 2013, Flamini et al., 2015, García-Herrera et al., 2013, Li et al., 2008, Ning et al., 2010, O’Dea and Nolan, 2012, Veljković et al., 2014). In addition, non-invasive, inverse computational methods, based on simultaneous acquisition of pressure gradients and diameters, (De Heer et al., 2012, Hamdan et al., 2012, Karatolios et al., 2013, Masson et al., 2008, Schlicht et al., 2013, Schulze-Bauer and Holzapfel, 2003, Smoljkić et al., 2015, Wittek et al., 2013, Zeinali-Davarani et al., 2011), are limited to describe the patient-specific behaviour during the cardiac cycle, but not at overload due to device expansion (Bosi et al., 2015, Bosi et al., 2016a, Bosi et al., 2016b).

Transcatheter aortic valve replacement (TAVR) is an established technique to treat severe aortic valve stenosis in high surgical risk patients (Zajarias and Cribier, 2009). TAVR is an ideal setting to advance the field of patient-specific modelling, as the substrate of the TAVR population, with highly calcified implantation sites, present fewer variations in terms of mechanical properties compared to other cardiovascular sites or patient groups (Pham et al., 2017). TAVR outcomes depend on appropriate patient assessment (Kalogeras, 2012, Ruparelia and Prendergast, 2015), and complications such as paravalvular leak (PVL) (Azadani et al., 2009, Tamburino et al., 2011) and onset of conduction abnormalities leading to permanent pacemaker implantation (Binder et al., 2013, Bleiziffer et al., 2010) remain common, therefore warranting a patient-specific computational approach to enhance patient selection (Schoenhagen et al., 2011, Taylor and Figueroa, 2009, Vy et al., 2015). A few patient-specific computational models are already available in the literature for TAVR (Bianchi et al., 2016, Capelli et al., 2012, Gunning et al., 2014, Morganti et al., 2014, Sirois et al., 2011, Sturla et al., 2016, Wang et al., 2015, Wu et al., 2016), but choice of the material parameters for the implantation site remains open (Tseng et al., 2013).

The aim of this work was the development of a computational framework for TAVR simulations, which included patient-specific anatomical site and population-specific mechanical response, based on a retrospective clinical study. The implantation site material parameters were adjusted in order to minimise the error between computational prediction and clinical results in terms of implanted stent diameter as measured from post-procedural fluoroscopy images. The finally obtained computational model had increase accuracy and reliability for prospective future clinical applications.

## 2 Materials and methods

Pre-procedural clinical images from a selected TAVR population were processed to create patient-specific finite element (FE) models and simulate the intervention. Post-implantation fluoroscopy images were used to tune the material properties of the FE implantation site model and echocardiography images to validate the computational results with clinical outcomes. The FE analyses were performed using Abaqus 6.14/Explicit (Dassault Systèmes Simulia Corp., Providence, RI, USA) under the hypothesis of quasi-static conditions.

### 2.1 Patient population and image analysis

Fourteen patients (age at intervention = 79.3 ± 8.0 years, 9 males; Table 1), who underwent successful TAVR with the Edwards Sapien XT device at the Heart Hospital (London, UK) between October 2013 and November 2014 were retrospectively selected for this study. One patient received the 23 mm device, nine the 26 mm and four the 29 mm. In all patients, the Sapien XT device was implanted in sub-coronary position, a third below the annulus of the native aortic valve (AV) according to guidelines (Cribier et al., 2009).

**Table 1:** Patients selected for the study. In the last column of the table, the clinical outcome in terms of paravalvular leakage (PVL) is reported. RCC = right coronary cusp; LCC = left coronary cusp; NCC = non-coronary cusp.

Patient	Age at TAVR	Gender	Sapien XT device [mm]	Post-TAVR fluoroscopy diameter [mm]	Paravalvular leakage
1	85	M	29	25.3	trivial in NCC
2	77	M	29	27.1	trivial in NCC
3	78	M	26	26.3	trivial in NCC
4	59	F	26	23.2	trivial in RCC
5	69	F	26	23.7	1 trivial jet in RCC-LCC, 1 trivial jet in NCC
6	76	M	26	25.0	No PVL
7	85	M	26	25.1	trivial in RCC, almost absent
8	78	M	26	25.4	1 mild jet in RCC, 1 trivial jet in NCC
9	88	F	23	21.4	1 trivial jet LCC-NCC, 1 mild+ jet RCC-NCC
10	83	M	29	25.5	trivial in NCC-RCC
11	78	F	26	22.7	trivial in RCC-LCC
12	83	M	26	25.6	trivial
13	81	M	29	26.4	1 mild jet in RCC, 1 trivial jet in RCC-NCC
14	90	F	26	23.5	No PVL

**Fig. 1:**
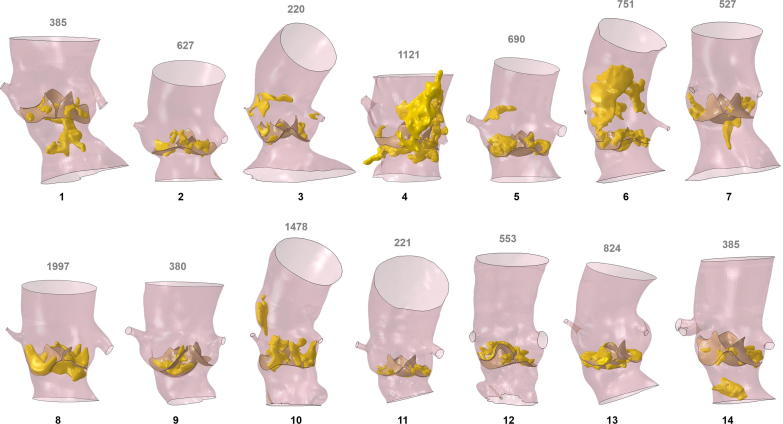
3D anatomical reconstructions from CT scans of the 14 patients considered in this study; the calcific plaques on both leaflets and vessel are represented in yellow, whilst the number at the top represents the volume of calcium in the leaflets only [mm^3^]. (For interpretation of the references to colour in this figure legend, the reader is referred to the web version of this article.)

Pre-procedural computed tomography (CT) images were post-processed (Mimics, Materialise Inc., Leuven, Belgium, (Schievano et al., 2007)) to create 3D anatomical models of the implantation sites including left ventricular outflow tract (LVOT), aortic root, ascending aorta, aortic leaflets, coronary arteries and valve/vessel calcifications (Fig. 1). The volume of calcific deposits in the valve was quantified from the CT 3D reconstructions.

The stent expansion diameter was measured from the post-implantation fluoroscopy images – acquired in a plane parallel to the axis of the stent in a lateral projection – at valve level. Although circular cross-section can be assumed for the Sapien device deployed by means of a high pressure balloon (Tseng et al., 2013), the computational model was first reoriented in the same projection as the fluoroscopy images, before measuring the projected distance at the level of the TAVR valve for comparison. PVL was assessed immediately post-TAVR by echocardiography and was present in twelve cases – nine trivial and three mild. The position of the jet was evaluated by dividing the aortic valve cross-section in thirds according to the valve cusp positions (right coronary, left coronary and non-coronary cusp).

### 2.2 TAVR FE model

The aortic roots and leaflets reconstructed from CT were meshed with 4-node shell general-purpose elements with reduced integration (1 mm average size) after sensitivity analysis (Finotello et al., 2017), whilst the calcific plaques were discretised with 4-node tetrahedral elements (0.5 mm average size). The implantation sites were constrained at their distal and proximal extremities to avoid rigid motion. Tie constraints were applied between the inner aortic surface and the external edge of the leaflets. The same constraints were applied to the calcific deposits and their respective leaflets or the ascending aortic wall when present.

Literature data from experimental tests of *ex-vivo* specimens (Billiar and Sacks, 2000, Dunmore-Buyze et al., 2002, Durmaz et al., 2010, Grande et al., 1998, Hamdan et al., 2012, Maleki et al., 2014, Walraevens et al., 2008, Weinberg et al., 2009) (Table 2) were used to set-up the initial implantation site material model, considering the characteristics of the TAVR patient population: old age, highly calcified and stiff implantation site, with no visible deformations during the cardiac cycle. Considering that the mechanical response to stent implantation depends on the arterial tissue, but also on the surrounding structures (Kim et al., 2013) and that the contribution of the two cannot be discerned from *in vivo* data, a simple linear elastic law was adopted rather than a more realistic and complex description (heterogeneous, non-linear, anisotropic) (Gasser et al., 2006, Holzapfel and Gasser, 2001), for both arterial wall and leaflets, with the stiffest range of available properties as the most representative for TAVR patients: E_artery_ = 22.6 MPa (Hamdan et al., 2012) and E_leaflets_ = 8.75 MPa (Weinberg et al., 2009). The same considerations led to the choice of tissue thicknesses (t): t_artery_ = 2.8 mm (Dunmore-Buyze et al., 2002) and t_leaflets_ = 2 mm (Grande et al., 1998).

**Table 2:** Material parameters.

	Young modulus [MPa]	Poisson’s ratio	Yield stress [MPa]	Density [kg/m^3^]	Thickness [mm]
Artery	3–22.6 (Durmaz et al., 2010, Hamdan et al., 2012, Walraevens et al., 2008)	0.45	–	1250 (Weinberg et al., 2009)	1–2.8 (Dunmore-Buyze et al., 2002, Walraevens et al., 2008)
Leaflets	4–8.75 (Billiar and Sacks, 2000, Maleki et al., 2014, Weinberg et al., 2009)	0.45	–	1250 (Weinberg et al., 2009)	0.5–2 (Billiar and Sacks, 2000, Grande et al., 1998)
Calcium	0.2–60,000 (Ebenstein et al., 2009, Halevi et al., 2015, Loree, 1994, Morlacchi et al., 2014, Wang et al., 2012, Weinberg et al., 2009)	0.3 (Gastaldi et al., 2010)	0.4 (Gastaldi et al., 2010)	2000	
MP35N	232,800	0.3	414	8000	
PET	600 (Tzamtzis et al., 2013)	0.4	–	1380	0.06

For calcific deposits, the Young’s moduli reported in literature are extremely variable, ranging from 0.2 to 60,000 MPa (Ebenstein et al., 2009, Halevi et al., 2015, Loree, 1994, Morlacchi et al., 2014, Wang et al., 2012, Weinberg et al., 2009), and only a few studies refer specifically to calcific deposits in the aortic leaflets (Halevi et al., 2015, Weinberg et al., 2009). An elasto-plastic model with perfect plasticity was adopted to simulate fracture, as it was assumed that after yielding the material had no resistance, with an initial Young’s modulus = 400 MPa, yielding stress = 0.4 MPa and Poisson’s ratio = 0.3 (Gastaldi et al., 2010).

Device geometries were generated from micro-CT scans (Metris X-Tek HMX ST 225 CT, Nikon Metrology, Belgium). The zigzag elements and vertical bars of the stents were meshed using beam elements with a rectangular section profile (0.6 mm radial thickness and 0.38 mm circumferential width), whilst a circumferentially wider rectangular section was assigned to the larger bars (1.15 mm circumferential width). After sensitivity analysis, the average length of the beam elements was 0.7 mm. The stent cobalt-chromium alloy (MP35N) was modelled as a homogeneous, isotropic, elasto-plastic material (Table 2). The biological valve mounted into the TAVR device was neglected in the FE model as the interaction between the stent and the implantation site was the focus of this study (Bailey et al., 2016).

The balloon used to perform balloon valvuloplasty (BAV) just before the TAVR procedures and to expand the Sapien XT device, is a non-compliant PET balloon, with nominal inflation pressure of 5 atm (0.507 MPa). The balloon was designed in the expanded configuration and meshed with 4-node membrane elements (average size 0.5 mm in the longitudinal direction and 0.38 mm in the circumferential direction). PET was described as a homogeneous, isotropic, linear-elastic material (Table 2).

A general contact algorithm was adopted between the different parts of the system with hard contact property. A preliminary simulation was carried out to open the central portion of the aortic leaflets and allow insertion of the balloon and device. The balloon and stent models were placed coaxially to the patient-specific implantation site models. The balloon, constrained at its distal and proximal ends in circumferential and radial direction in order to mimic the bond to the catheter, and in circumferential and longitudinal directions at the central circumference to avoid rigid motion, was deflated to allow insertion into the patient-specific implantation sites and replicate BAV by inflating the balloon to nominal pressure, and deflating it again. The stent models were then crimped onto the balloon to the size of the delivery catheters using radial displacements applied to a coaxial cylindrical surface (surface elements, average size 0.5 mm). The stent expansion was simulated by inflating and deflating the balloon as done for BAV.

**Fig. 2:**
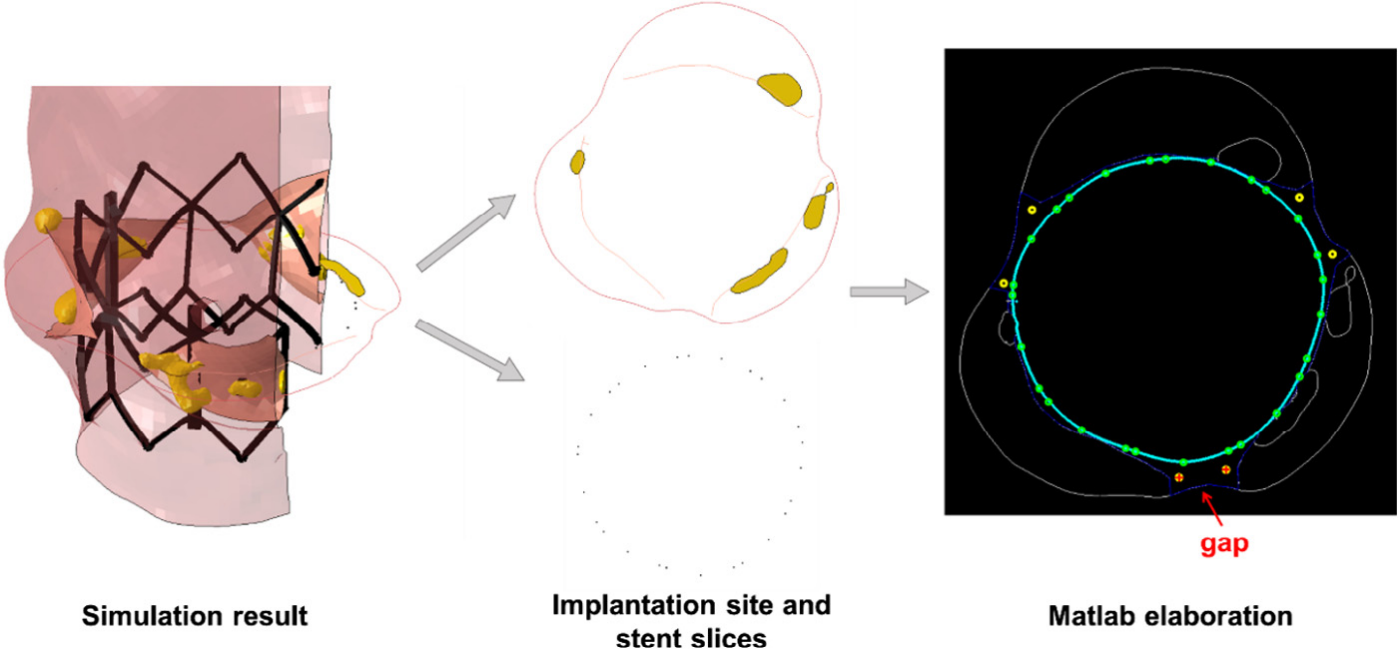
The left panel shows the results of the computational simulation for patient 11. From this 3D representation, 2D slices are captured separately for the implantation site (top section of the central panel) and for the stent (bottom section of the central panel). The slices are converted to black & white in Matlab, combining the information together (right panel): the inner portion of the implantation site, which will be subsequently used for all calculations is depicted in blue; the stent strut centroids are represented by green dots, whilst the interpolating spline in light blue; the yellow dots represent the maxima of the radial distance between stent and implantation site in this cross section; the red crosses show only the maxima indicating a continuous gap along the whole length of the stent. (For interpretation of the references to colour in this figure legend, the reader is referred to the web version of this article.)

A previously described Matlab (MatWorks, MA, US) function (Bosi et al., 2015) allowed quantification of the interaction between the device and the implantation site as a surrogate measure for PVL. The post-TAVR FE model was cut along the length at every 0.5 mm, and the cross-sectional images were analysed to identify the areas lacking contact. Only continuous gaps along the length of the stent were then considered to indicate potential PVL (Fig. 2). Position of PVL jets from the available echocardiography images was compared to the contact gaps in the corresponding computational cross-section.

### 2.3 Statistical analysis of aortic root material parameters

A statistical sensitivity analysis was performed to assess the influence of the unknown material parameters adopted to describe the implantation site model on the simulation results using a design of experiments (DOE) approach (Design-Expert software 10, Stat-Ease, Inc. Minneapolis, USA). DOE allows estimation of the effects of the variation of one or more factors on single or multiple output responses and determine which factors have a significant effect on the response. In this study, a two-level fractional factorial design was adopted to investigate the main effects and/or interaction effects of six input factors (E_artery_, t_artery_, E_leaflet_, t_leaflets_, E_calcium_ and Yield_calcium_) run at two levels each (minimum and maximum values from literature data) on one outcome output response (the stent diameter after balloon deflation). The material and thickness parameters initially adopted for the implantation site FE model were used to set the upper level for the two-level fractional factorial experiment, while the range identified from the literature was chosen to set the lower level, i.e. E_artery_ = 3 MPa, t_artery_ = 1 mm (Walraevens et al., 2008), E_leaflets_ = 4 MPa (Weinberg et al., 2009), t_leaflets_ = 0.5 mm (Billiar and Sacks, 2000). For the calcific deposits the minimum value were chosen as E_calcium_ = 100 MPa (Ebenstein et al., 2009), and Yield_calcium_ = 0.1 MPa (Gastaldi et al., 2010).

Patient 13 was selected as considered representative of the TAVR population, with average degree of calcification (824 mm^3^, compared to the average of 726 ± 503 mm^3^) and without particularly irregular anatomy; moreover, in the first run of simulations with the initial material properties, patient 13 had a diameter difference between simulated and actual value of −5.6%, the closest to the average error for the population.

Sixteen simulations were performed, with different combinations of input values, as indicated by DOE, with resolution IV (Saleem and Somá, 2015). A Pareto Chart was used to display the standardised effect (t-value) of each input term, i.e. factor or combination of factors, on the outcome parameter. T-values above the Bonferroni limit identified effects with almost certain influence, whilst those above the t-value limit indicated effects with possible influence. Analysis of Variance (ANOVA) was performed to assess the effects of the factors and factorial interactions on the output response and refine the values of only the significant factors. The results of the statistical analysis led to a new set of material parameters that were implemented in the FE model to re-run the patient-specific simulations. The new computational results were then compared with the diameters measured from fluoroscopy images and with echocardiography clinical outcomes.

## 3 Results

**Fig. 3:**
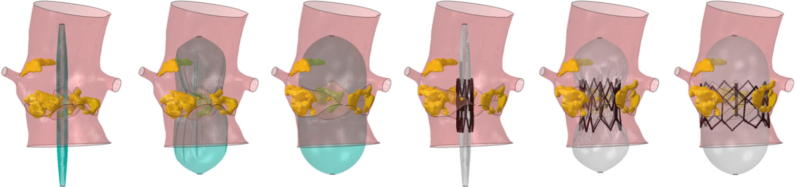
Simulation of valvuloplasty and subsequent Sapien XT 26 mm implantation for patient 5.

Patient-specific FE simulations were successfully completed for all 14 cases; Fig. 3 shows an example of the expansion phases of the valvuloplasty balloon and of the stent-balloon system.

**Fig. 4:**
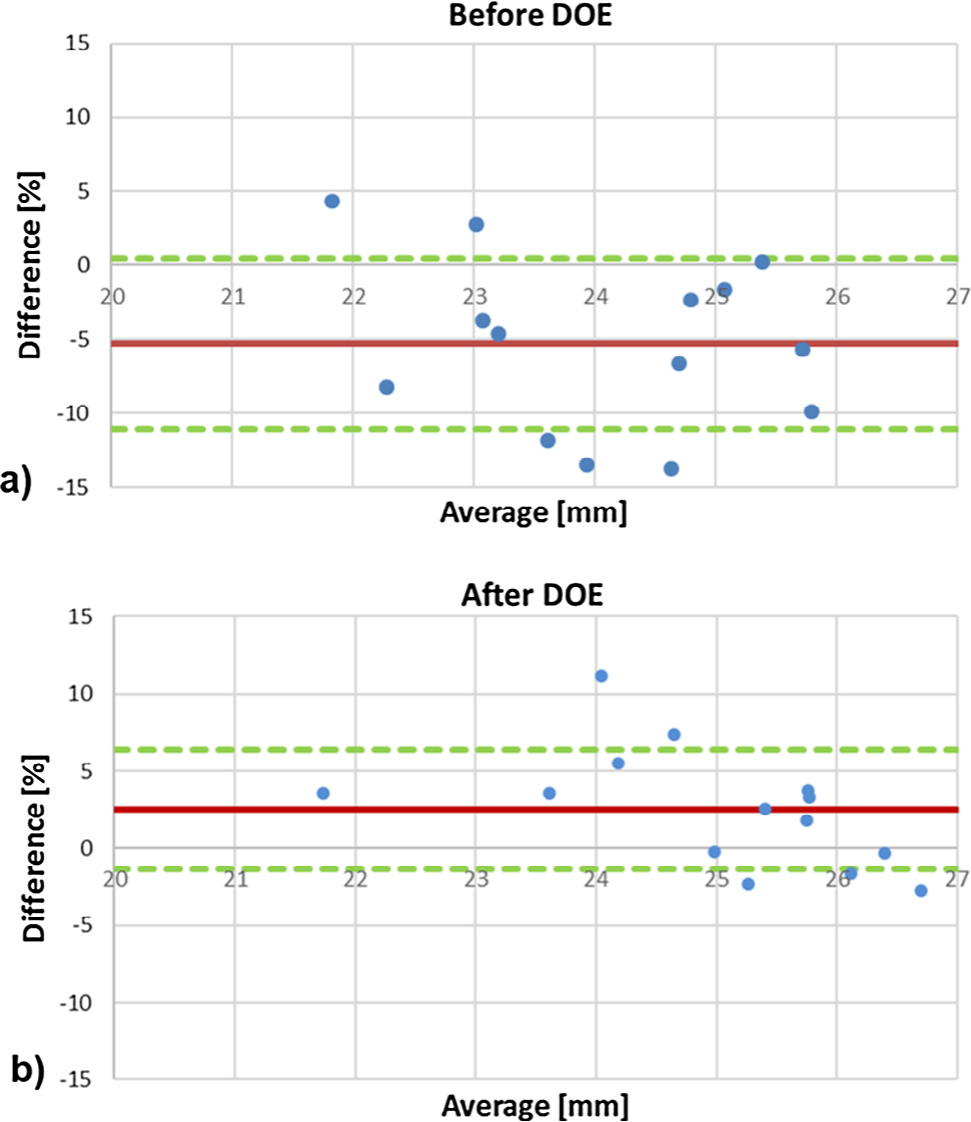
Bland Altman plots for the comparison between stent diameters measured from fluoroscopy images and computational results before (a) and after (b) refinement of the material parameters: the red line represents the mean difference and the green dashed lines ± standard deviations. (For interpretation of the references to colour in this figure legend, the reader is referred to the web version of this article.)

In the first set of simulations, with the stiffest material properties and highest thicknesses used to describe the implantation site, the average diameter at the end of balloon deflation was 23.4 ± 1.3 mm. Compared to the fluoroscopy image measurements (average diameter 24.7 ± 1.6, Table 1), there was a mean difference of −5.3 ± 5.7%, with maximum error of −13.8% recorded for patient 3. The Bland Altman plots (Fig. 4a) show that most FE stents were under-expanded compared to the clinical counterparts, thus suggesting that the material chosen for the TAVR implantation site was too stiff.

**Fig. 5:**
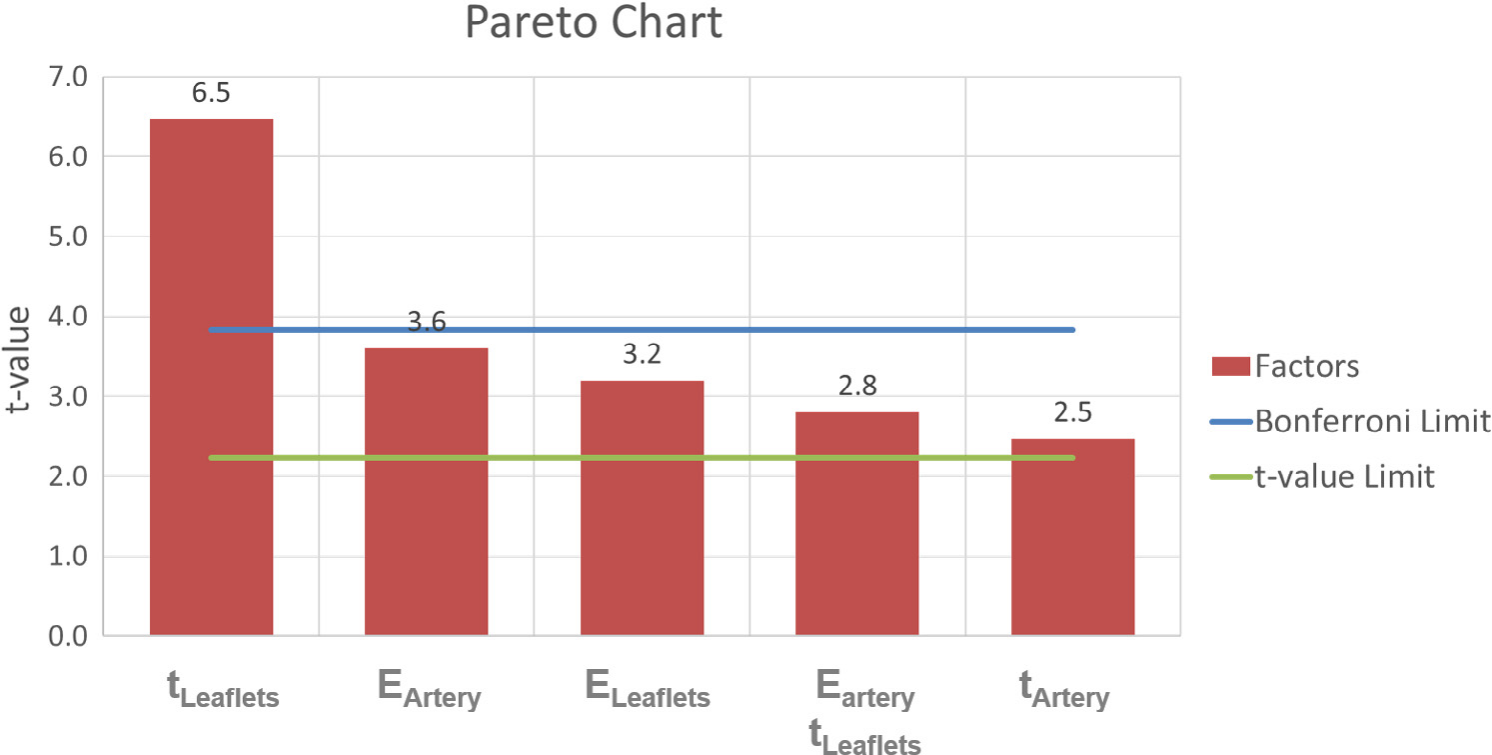
Pareto Chart showing the magnitude of the t-value for the significant effect analysed.

The Pareto chart for patient 13 shows the t-value for each effect (Fig. 5), including factors and interaction of factors, where the Bonferroni limit was 3.8 and t-value limit was 2.2. The most significant factor was the leaflet thickness, followed by the Young’s modulus of the arterial wall and the leaflets, the combination of the first two, and the arterial thickness (Table 3). Standard error was 0.15 for every factor. The model F-value was 15.93 implying statistical significance, i.e. 0.02% chance that an F-value this large could occur due to noise.

**Table 3:** F-ratio, *p value*, regression coefficient and t-value for the significant factor and the most significant interaction.

Factors	F-ratio	*p value*	Regression coefficient	t-value
E_artery_	13.05	.0048	−0.54	−3.60
t_artery_	6.08	.0333	−0.37	−2.47
E_leaflets_	10.23	.0095	−0.48	−3.20
t_leaflets_	42.36	<.0001	−0.97	−6.47
E_artery_ t_leaflets_	7.93	.0183	−0.42	−2.80

Given the DOE results, a new set of refined material parameters was found (Table 4) and FE analyses for all patients were run again accordingly. The stent diameter % difference was 2.53 ± 3.88% with nine cases of over-expansion and five cases of under-expansion, thus centering the distribution (Fig. 4b). The maximum over-expansion error was 11.2% (patient 11) and the maximum under-expansion error −2.7% (patient 2).

**Table 4:** Population-specific material parameter for the TAVR anatomical implantation site after DOE analysis.

TAVR population-specific material parameters	
E_artery_	7.78 MPa
t_artery_	1.9 mm
E_leaflets_	6.375 MPa
t_leaflets_	0.5 mm
E_calcium_	250 MPa
Yield_calcium_	0.25 MPa

**Fig. 6:**
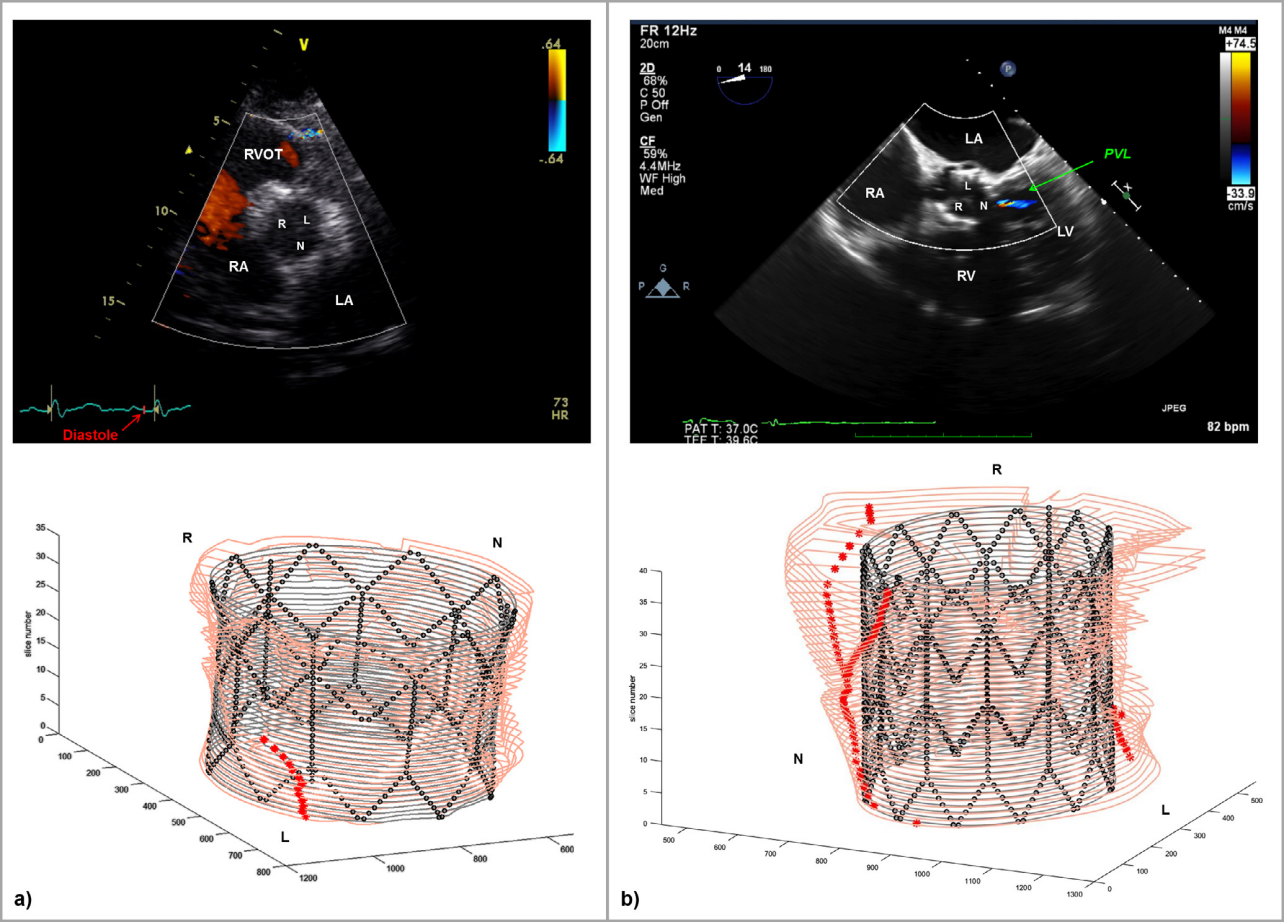
(a) In the top panel, transthoracic echocardiographic parasternal short axis view of the aortic valve for patient 14: it is not possible to appreciate any paravalvular regurgitation. The ECG shows that the image was taken during diastole, i.e. during the bioprosthetic valve closure. In the bottom panel, Matlab elaboration of the FE results, confirming the absence of PVL. (b) In the top panel, transthoracic echocardiographic 5 chambers view of the aortic valve for patient 2: a single jet of paravalvular regurgitation is showed in the non-coronary cusps of the valve. The green arrow points at the PVL jet. In the bottom panel, Matlab elaboration of the FE results, confirming the presence of PVL in the same position. RVOT = right ventricular outflow tract, LV = left ventricle, RV = right ventricle, RA = right atrium; LA = left atrium; R = right coronary cusp; L = left coronary cusp; N = non-coronary cusp. (For interpretation of the references to colour in this figure legend, the reader is referred to the web version of this article.)

Two examples are reported in the figure to highlight PVL detection: in patient 14, the algorithm did not find any continuous gap along the length of the virtually implanted stent (Fig. 6a); a partial gap is highlighted by the red asterisks in the proximal portion, but is interrupted in the middle portion of the stent. Indeed, post-TAVR echocardiography did not show PVL. On the contrary, for patient 2 (Fig. 6b), the post-TAVR implantation echocardiography highlighted one trivial jets of PVL in the non-coronary cusp (NCC). The corresponding Matlab graph showed two channels starting from the distal portion of the stent and coming together in one towards NCC. Overall, the computational framework correctly indicated presence/absence of PVL in 79% cases (n = 11) – 4 under-expanded and 7 over-expanded. The other three patients (4, 11 and 12) presented a trivial jet at echocardiographic examination, with patient 11 showing the largest error in terms of stent diameter prediction (11.2%). All patients who did not have PVL were correctly identified by the code.

PVL location was correctly recognized in six (patients 1, 2, 3, 9, 10, 13) over the nine PVL patients appropriately identified (67% of cases).

## 4 Discussion

In this work, a computational framework for TAVR implantation of the Edwards Sapien XT device was developed and tested in 14 retrospective cases. The implantation site computational model was designed based on the patient-specific anatomy and refined in terms of material properties to replicate the TAVR population mechanical response to device implantation using clinical data.

Patient aortic root and calcified leaflet morphology can nowadays be retrieved from pre-assessment CT. However, information on each patient mechanical response to device expansion cannot be extracted from *in vivo* pre-procedural clinical investigations and literature data from *ex-vivo* experimental tests are usually adopted in computational models. To further refine the selection of the material properties of the different biological structures (arterial wall, aortic leaflets and calcific deposits) for the specific TAVR population, elderly patients with severe aortic stenosis, we first initialized the FE model with the stiffest and thickest data available from literature. Then, a statistical approach conventionally used in engineering to optimise product design was adopted for the first time in this context to investigate the main effects of the six unknown material parameters used to describe the implantation site in a selected patient, considered representative of the entire cohort of patients. It would be interesting to run this DOE analysis on a set of many TAVR cases to quantify the differences from the optimized parameters found and to simultaneously optimize the material for more than one case, thus hopefully reducing even more the error from clinical measurements. The leaflet thickness was the most significant factor affecting the computational results, followed by the Young’s modulus of the artery and the leaflet.

A second set of simulations for all patients using the refined material parameters for the implantation site resulted in lower differences between the computational results and the clinical stent diameters, thus showing the outcomes of the optimisation process. The population-specific material parameters identified in this study for this population of TAVR cases will be further tested in the future on a larger set of patients to capture potential further variability and prove reliability also using different devices.

A post-processing Matlab code was used to automatically analyse the computational results in terms of geometric interaction between implanted TAVR stent and deformed implantation site. Based on purely geometric information, it was possible to derive some considerations on the potential development of jets of PVL, on the hypothesis that PVL is caused by a suboptimal apposition of the device onto the anatomical site. The algorithm was able to capture the presence/lack of PVL in 79% of patients, thus attesting its specificity. Moreover, the code was able to identify PVL jets location origin in 67% of cases. In clinical practice, PVL severity is assessed with measurements from echo colour Doppler (Sinning et al., 2013) in a scale from trivial, mild, moderate to severe, depending on regurgitant jet dimensions and length, during the diastolic phase. This quantification is technically challenging and highly operator dependent as different cross-sectional view of the device might result in highly different degrees of PVL, both in terms of severity, and in terms of position. With the purposely developed code, we aimed to provide an objective quantification of PVL, derived purely from geometrical consideration; although this parameter is difficult to quantify in clinic, the location was considered recognized if the code found the jet in the same third of the aortic valve, since no more precision is achievable from echocardiography images. It has to be underlined that the computational results provided merely static geometrical information about the interaction stent-implantation site, while the PVL jet might move during the diastolic phase, thus making even more difficult for the operator to report its exact location. In the three cases in which the code inappropriately identified PVL, there was over-expansion (two), but also one case of under-expansion of the stent model compared to the actual diameter. Therefore, there is no clear association between the ability to predict PVL and correct prediction of the implanted stent diameter.

In terms of limitations for this study, the first consideration concerns the measurements from fluoroscopy images, prone to calibration errors and on the assumption of cross-sectional circularity. Simultaneous biplane fluoroscopy images could improve the measurement acquisition and would allow 3D reconstruction of the stent geometry *in situ* through back projection (Cosentino et al., 2014). DOE was carried out on a selected patient: this could be repeated for other patients to test the parameter settings of the calcified aortic root/LVOT derived from the patient cohort, and average the values of the parameters to minimise further the errors.

In the specific clinical setting of severe aortic valve stenosis, a population-specific approach to model the mechanical response of the implantation site to device deployment was considered acceptable as small variations are present in this groups of patients. In the future, advanced image modalities combined with computational modelling may allow for further personalisation of the model.

Additional computational fluid dynamic analysis could help study the local flow conditions and quantify the severity of PVL. In the future, refinements of the Matlab algorithm will improve identification of PVL location and introduce a measure for the degree of regurgitation by analysing gap areas and geometrical complexity. The methodology, if successfully validated, would allow the evaluation of PVL severity without using additional high-computational-cost analyses.

Simplicity and speed of computation have been the main drives for the model here developed, which is not meant to derive accurate localised stress/strain information in the arterial wall/stent, but is designed to provide fast, clinically meaningful predictive information (e.g. stent diameter and possible onset of PVL) built from routinely acquired clinical diagnostic data. The small discrepancy between the computational results and the clinical measurements achieved with the described model in this specific patient population, demonstrates that, despite simple, the computational framework could be used to this purpose.

## 5 Conclusions

In this work, we developed a patient-specific computational framework to virtually simulate TAVR procedures. Two main objectives were achieved: the tuning of a set of material/thickness parameters able to describe the implantation site response to TAVR for the TAVR patient population, and the validation of the numerical model over a small cohort of patients. This computational framework could be used on one side to aid the design and test of new TAVR devices in validated implantation sites, and, on the other, to enhance the assessment of patients selected for TAVR.
